# Supplementary material for: The SARS-CoV-2 Alpha variant exhibits comparable fitness to the D614G strain in a Syrian hamster model
Source: Commun Biol. 2022 Mar 10;5:225. doi: 10.1038/s42003-022-03171-9 (PMC8913834; doi:10.1038/s42003-022-03171-9)
Supplement: Supplementary file 1 — Supplementary Information [file 42003_2022_3171_MOESM1_ESM.pdf]

Title: The SARS-CoV-2 Alpha variant exhibits comparable fitness to the D614G strain in a Syrian hamster model

Author list: Maxime Cochin*[1], Léa Luciani*[1], Franck Touret[1], Jean-Sélim Driouich[1], Paul-Rémi Petit[1], Grégory Moureau[1], Cécile Baronti[1], Caroline Laprie[2], Laurence Thirion[1], Piet Maes[3], Robbert Boudewijns[4], Johan Neyts[4], Xavier de Lamballerie[1], Antoine Nougairède[1]

Affiliations:

[1] Unité des Virus Émergents (UVE: Aix-Marseille Univ-IRD 190-Inserm 1207), Marseille, France

[2] Laboratoire Vet-Histo, Marseille, France

[3] KU Leuven Department of Microbiology, Immunology and Transplantation, Laboratory of Clinical and Epidemiological Virology, Rega Institute, Leuven, Belgium

[4] KU Leuven Department of Microbiology, Immunology and Transplantation, Laboratory of Virology and Chemotherapy, Rega Institute, Leuven, Belgium

* These authors contributed equally to this article.

Corresponding author: Antoine Nougairède (antoine.nougairede@univ-amu.fr)

Key words: SARS-CoV-2; B.1.1.7; B.1.351; B.1.167.2; Alpha variant; Beta variant; Delta variant; Preclinical; hamster model; transmission; replicative fitness; host response

Legends supplemental Figures and Tables:

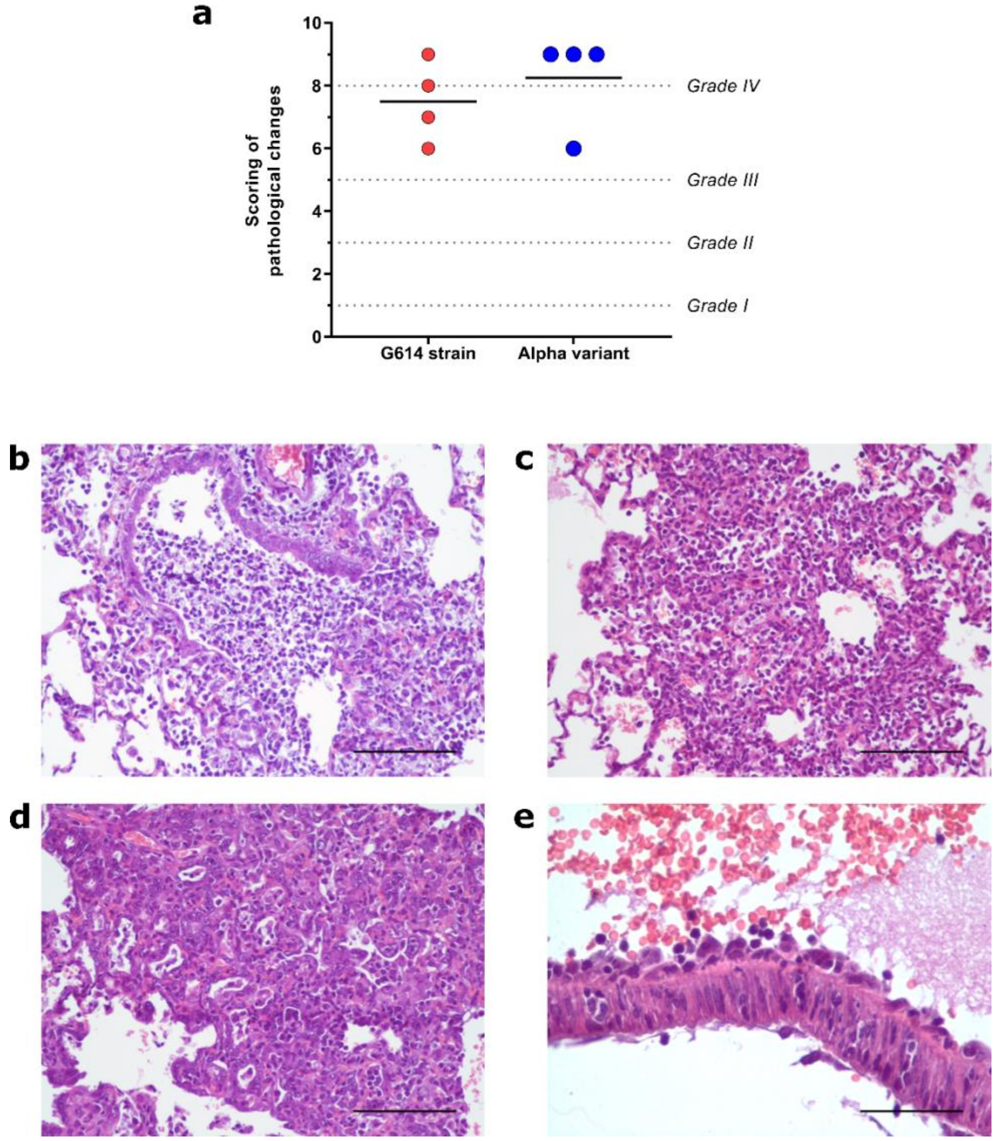

Supplemental figure 1: Lung histopathological changes induced by Alpha variant and G614 strain. Groups of 4 hamsters were intranasally infected with 10^4 $TCID_{50}$ of Alpha variant or G614 strain and sacrificed at 5 dpi. (a) Scoring of histopathological changes measured following criteria presented in Supplementary Tables 6 and 7, as previously described[1]. Rows represent the mean score of each group (n=4; details in Supplemental Table 8). Difference between both groups is non-significant (Mann-Whitney test). (b-e) Representative images of lesions observed in both animal groups. (b) Representative image of bronchitis: terminal bronchiole filled with degenerated neutrophils and necrotic cellular debris (scale bar = 100µm; animal of ‘G614 strain’ group). (c) Representative images of broncho-interstitial pneumonia: area of lung consolidation with interstitial inflammation, expansion of alveolar walls by inflammatory infiltrates (macrophages, heterophils) also filling alveolar lumina (scale bar = 100µm; animal of ‘Alpha variant’ group). (d) Representative images of broncho-interstitial pneumonia: area of lung consolidation showing interstitial inflammation (macrophages, heterophils) and alveolar septa lined by low cuboïdal epithelial cell (type II pneumocytes hyperplasia) (scale bar = 100µm; animal of ‘G614 strain’ group). (e) Representative images of vasculitis, arteriolar wall infiltration by leucocytes with cellular debris, subendothelial leucocytic collection (scale bar = 50µm; animal of ‘Alpha variant’ group).

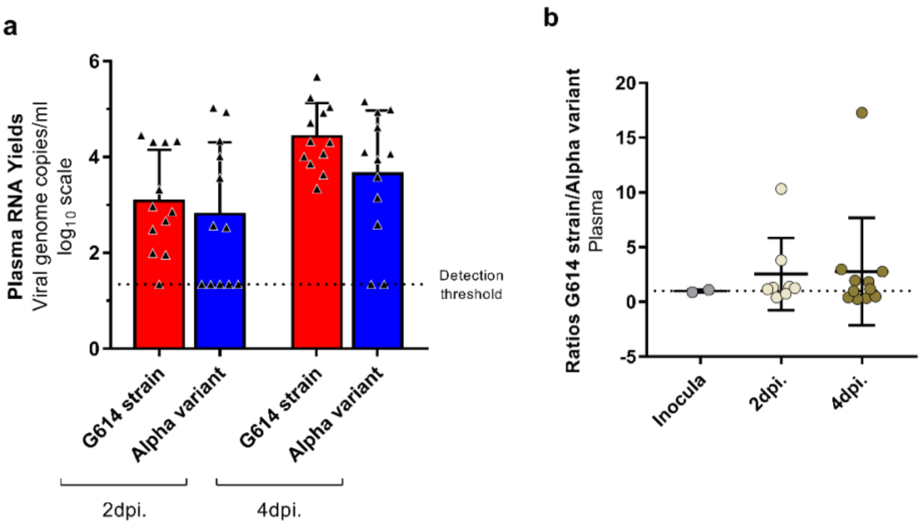

Supplemental Figure 2: Viral RNA yields in plasmas measured using a RT-qPCR assay. Results for (a) comparative assessment and (b) competition experiments (expressed as [G614/ Alpha] ratios). Two specific RT-qPCR assays were used to measure the quantity of each virus in plasmas. Rows represent mean ± SD of each group (n=10 to 11).

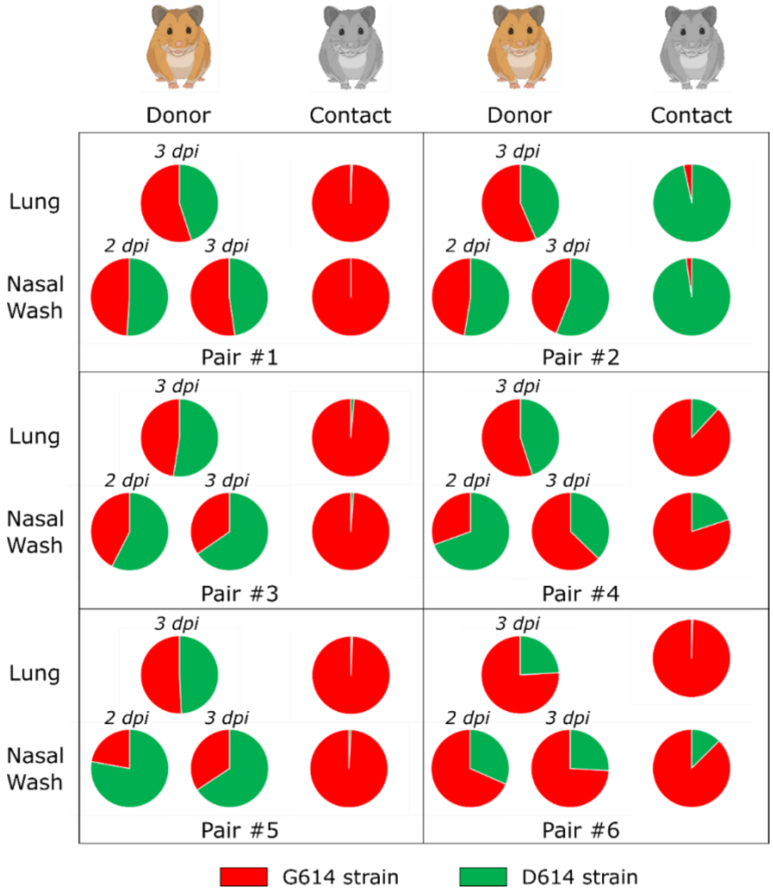

Supplemental Figure 3: Transmission experiment with G614 and D614 strains. A group of 6 hamsters, named 'donors', was intranasally infected with an equal proportion of both viral strains for competition experiment (total: 20 TCID$_{50}$). At 2 dpi, each donor was co-housed with a contact animal during a period of 6 hours and received a nasal wash. Donors and contacts were sacrificed at 3 dpi and at 3 dpc respectively. This figure is a graphical representation of the proportion of each virus found in

lungs and nasal washes for each pair of animals in transmission experimentations. Results were obtained using sequencing method described in the ‘Molecular biology’ part of the ‘Materiel and Methods’ section.

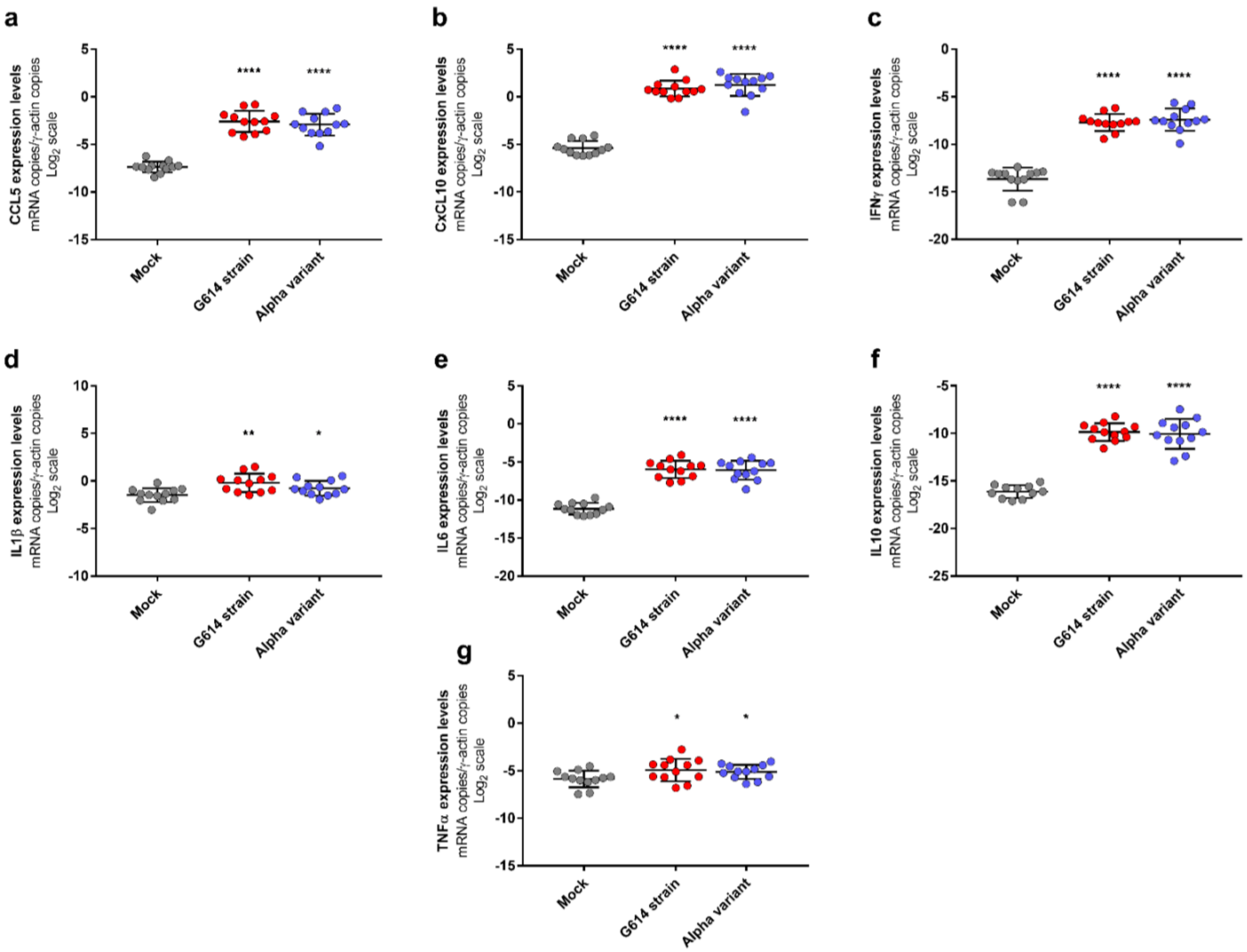

Supplemental Figure 4: Expression level of cytokines genes in the lungs at 4 dpi after infection with G614 strain or Alpha variant. A panel of seven cytokines is presented: Ccl5 (a), Cxcl-10 (b), Interferon-γ (c), IL-1β (d), IL-6 (e), IL-10 (f) and TNF-α (g). Gene expression was quantified using RT-qPCR assays expressed as mRNA copies/γ-actin copies. ***, ** and * symbols indicate that the mean expression for the G614 or for the Alpha group are significantly higher than those of the mock-infected group with a p-value ranging between 0.0001-0.001, 0.001–0.01, and 0.01–0.05, respectively (Mann-Whitney and unpaired t tests). Rows represent mean ± SD of each group.

Supplementary table 1: Statistical analysis of YRNT assays at 90% inhibition for comparison between viral strains used for seroneutralisation assay (p-values obtained using multiple unpaired t tests with Holm-Sidak method)

Comparaison between viral strains used for seroneutralisation assay	Viral strain used for animal infection	
	G614 strain	Alpha variant
G614 strain vs. Alpha variant	0.3975	0.1983
G614 strain vs. Beta variant	**0.0398**	**0.0450**
Alpha variant vs. Beta variant	**0.0020**	**0.0123**
G614 strain vs. Delta variant	0.6573	0.7471
Alpha variant vs. Delta variant	0.4060	0.2409
Beta variant vs. Delta variant	**0.0019**	**0.0080**

Supplementary table 2: Statistical analysis of YRNT assays at 90% inhibition for comparison between viral strains used for animal infection (p-values obtained using multiple unpaired t tests with Holm-Sidak method)

Comparaison between viral strains used for animal infection	Viral strain used for seroneutralisation assay			
	G614 strain	Alpha variant	Beta variant	Delta variant
G614 strain vs. Alpha variant	0.9945	0.0510	0.0746	0.3011

Supplementary table 3: Statistical analysis of YRNT assays at 99% inhibition for comparison between viral strains used for seroneutralisation assay (p-values obtained using multiple unpaired t tests with Holm-Sidak method)

Comparaison between viral strains used for seroneutralisation assay	Viral strain used for animal infection	
	G614 strain	Alpha variant
G614 strain vs. Alpha variant	0.2608	0.1687
G614 strain vs. Beta variant	0.1030	**0.0474**
Alpha variant vs. Beta variant	0.0794	**0.0016**
G614 strain vs. Delta variant	0.1637	0.9966
Alpha variant vs. Delta variant	0.3811	0.0934
Beta variant vs. Delta variant	0.0388	**0.0060**

Supplementary table 4: Statistical analysis of YRNT assays at 99% inhibition for comparison between viral strains used for animal infection (p-values obtained using multiple unpaired t tests with Holm-Sidak method)

Comparaison between viral strains used for animal infection	Viral strain used for seroneutralisation assay			
	G614 strain	Alpha variant	Beta variant	Delta variant
G614 strain vs. Alpha variant	0.4628	**0.0299**	0.3282	**0.0369**

Supplementary table 5: Evaluation of the quantification protocol for G614 and D614 proportions

Theoretical mix tested G614-D614	Theoretical ratios G614/D614	F2 primer		R1 primer		G614 Average of the proportions of the two primers	D614 Average of the proportions of the two primers	Estimated ratios G614/D614
		Proportion of G peak height*	Proportion of A peak height*	Proportion of C peak height*	Proportion of T peak height*			
0-100	0,00	0	1	0	1	0	1	0,00
10-90	0,11	0,134	0,886	0,153	0,864	0,143	0,875	0,16
30-70	0,43	0,302	0,679	0,314	0,668	0,308	0,674	0,46
50-50	1,00	0,517	0,452	0,440	0,484	0,478	0,468	1,02
70-30	2,33	0,704	0,268	0,727	0,256	0,715	0,262	2,73
90-10	9,00	0,845	0,072	1,000	0,065	0,923	0,068	13,47
100-0	100,00	1	0	1	0	1	0	100,00
Water	0,00	0	0	0	0	0	0	0,00
						(F2 'G peak' + R1 'C peak')/2	*(F2 'A peak' + R1 'T peak')/2*	*G614 Average/ D614 Average*

* adjusted peak height of variant A ÷ sum of the peak heights of variants A and B

Supplementary table 6: Semi-quantitative histopathological lung inflammation scoring system

Lesion	Description	Intensity	Score
**Interstitial pneumonia**	1 or 2 foci with 10-20 cells or small area with two-fold thickening of alveolar septa	Mild	1
	3 to 5 foci with 10-30 cells or widespread areas with two-fold thickening of alveolar septa	Moderate	2
	5 foci of 10-50 cells or widespread areas with two-fold or three-fold thickening of alveolar septa throughout the lung	Marked	3
	5 foci of 10-100 cells or widespread areas with three to fourfold-thickened alveolar septa throughout the lung	Severe	4
**Bronchitis**	1 or 2 bronchi section(s) filled with rare necrotic or apoptotic cells or rare inflammatory cells or partially surrounded by scarce inflammatory cells	Mild	1
	3 to 5 bronchi filled with necrotic cells or inflammatory cells or partially surrounded by a few inflammatory cells	Moderate	2
	6 to 10 bronchi filled with necrotic cells or inflammatory cells, partially or sub-completely surrounded by inflammatoty cells	Marked	3
	Numerous bronchi filled with inflammatory or cellular debris or completely surrounded by numerous inflammatory cells	Severe	4
**Vascular changes**	Absent	-	0
	Present	-	1
**Hemorrhagic necrosis**	Absent	-	0
	Focal to multifocal	Mild	1
	Coalescing to extensive necrosis	Severe	2

Supplementary table 7: Semi-quantitative histopathological lung inflammation grading system

Cumulative score	Grade	Broncho-interstitial pneumonia
0	0	Normal
1-3	I	Mild
4-5	II	Moderate
6-8	III	Marked
9-10	IV	Severe

Supplementary table 8: Hamster's weights and detailed scores of lung histological changes

Group	Animal ID	Weights (g)						Histological scores					
		Day of infection	Day +1	Day +2	Day +3	Day +4	Day +5	Interstitial pneumonia	Bronchitis	Vascular changes	Hemorrhagic necrosis	Total score	Grade
G614 strain	**620**	48.9	49.0	45.9	46.4	44.6	45.4	4	4	1	0	9	4
	**621**	62.4	64.0	60.3	57.6	54.1	53.6	4	3	1	1	9	4
	**622**	58.8	58.5	53.4	49.9	47.2	44.8	4	3	1	1	9	4
	**623**	62.7	64.8	63.2	63.5	65.8	66.5	3	2	1	0	6	3
Alpha variant	**626**	64.1	65.7	63.4	59.3	57.2	54.1	3	3	1	0	7	3
	**627**	66.3	70.5	68.9	70.9	69.8	66.6	4	3	1	0	8	3
	**628**	66.3	69.0	66.2	60.8	59.0	57.0	4	4	1	0	9	4
	**629**	60.5	62.8	60.1	61.5	62.4	61.1	3	3	0	0	6	3

Supplementary references

1. Driouich, J.-S. *et al.* Favipiravir antiviral efficacy against SARS-CoV-2 in a hamster model. *Nat. Commun.* **12**, 1735 (2021).
