# Supplementary material for: The SARS-CoV-2 Alpha variant exhibits comparable fitness to the D614G strain in a Syrian hamster model
Source: Commun Biol. 2022 Mar 10;5:225. doi: 10.1038/s42003-022-03171-9 (PMC8913834; doi:10.1038/s42003-022-03171-9)
Supplement: Supplementary file 2 — Description of Additional Supplementary Files [file 42003_2022_3171_MOESM2_ESM.pdf]

Description of Additional Supplementary Files

File name: Supplementary Data 1
Description: Comparison experiment (Figure 1.b-g and Supplementary Figure 2.a)
